# Plasma microRNA Profiles as a Potential Biomarker in Differentiating Adult-Onset Still's Disease From Sepsis

**DOI:** 10.3389/fimmu.2018.03099

**Published:** 2019-01-11

**Authors:** Qiongyi Hu, Wen Gong, Jieyu Gu, Guannan Geng, Ting Li, Rui Tian, Zhitao Yang, Haocheng Zhang, Lingyun Shao, Tingting Liu, Liyan Wan, Jinchao Jia, Chengde Yang, Yi Shi, Hui Shi

**Affiliations:** ^1^Department of Rheumatology and Immunology, Ruijin Hospital, Shanghai Jiao Tong University School of Medicine, Shanghai, China; ^2^Department of Rheumatology and Immunology, The First People's Hospital of Yancheng Affiliated with Nantong University, Yancheng, China; ^3^Department of Rheumatology, Renji Hospital, Shanghai Jiao Tong University School of Medicine, Shanghai, China; ^4^Department of Critical Care Medicine, Ruijin Hospital, Shanghai Jiao Tong University School of Medicine, Shanghai, China; ^5^Department of Emergency, Ruijin Hospital, Shanghai Jiao Tong University School of Medicine, Shanghai, China; ^6^Department of Infectious Diseases, Huashan Hospital, Fudan University, Shanghai, China; ^7^Key Laboratory of Systems Biomedicine (Ministry of Education) and Collaborative Innovation Center of Systems Biomedicine, Shanghai Center for Systems Biomedicine, Shanghai Jiao Tong University, Shanghai, China

**Keywords:** adult-onset still's disease, AOSD, sepsis, microRNAs, biomarker, inflammation

## Abstract

Adult-onset Still's disease (AOSD) is a systemic inflammatory disease characterized by cytokine storm. However, a diagnostic test for AOSD in clinical use is yet to be validated. The aim of our study was to identify non-invasive biomarkers with high specificity and sensitivity to diagnosis of AOSD. MicroRNA (miRNA) profiles in PBMC from new-onset AOSD patients without any treatment and healthy controls (HCs) were analyzed by miRNA deep sequencing. Plasma samples from 100 AOSD patients and 60 HCs were used to validated the expression levels of miRNA by qRT-PCR. The correlations between expression levels of miRNAs and clinical manifestations were analyzed using advanced statistical models. We found that plasma samples from AOSD patients showed a distinct miRNA expression profile. Five miRNAs (miR-142-5p, miR-101-3p, miR-29a-3p, miR-29c-3p, and miR-141-3p) were significantly upregulated in plasma of AOSD patients compared with HCs both in training and validation sets. We discovered a panel including 3 miRNAs (miR-142-5p, miR-101-3p, and miR-29a-3p) that can predict the probability of AOSD with an area under the receiver operating characteristic (ROC) curve of 0.8250 in training and validation sets. Moreover, the expression levels of 5 miRNAs were significantly higher in active AOSD patients compared with those in inactive patients. In addition, elevated level of miR-101-3p was found in AOSD patients with fever, sore throat and arthralgia symptoms; the miR-101-3p was also positively correlated with the levels of IL-6 and TNF-α in serum. Furthermore, five miRNAs (miR-142-5p, miR-101-3p, miR-29c-3p, miR-29a-3p, and miR-141-3p) expressed in plasma were significantly higher in AOSD patients than in sepsis patients (*P* < 0.05). The AUC value of 4-miRNA panel (miR-142-5p, miR-101-3p, miR-29c-3p, and miR-141-3p) for AOSD diagnosis from sepsis was 0.8448, revealing the potentially diagnostic value to distinguish AOSD patients from sepsis patients. Our results have identified a specific plasma miRNA signature that may serve as a potential non-invasive biomarker for diagnosis of AOSD and monitoring disease activity.

## Introduction

Adult-onset Still's disease (AOSD) is a rare systemic inflammatory disease which affects multiple organs and systems. It's characterized by a high spiking fever, evanescent skin rash, sore throat, and arthralgia (1–3). Laboratory findings include neutrophilia, high levels of ferritin, elevated erythrocyte sedimentation (ESR) and C-reactive protein (CRP). For the AOSD etiology, the cytokine storm activated by innate immune cells plays a crucial role in the pathogenesis. These innate immune cells release pro-inflammatory molecules, including IL-1β, IL-6, IL-18, TNF-α, and macrophage inhibitory factors (4, 5). AOSD has been studied for almost half a century since first described by Bywaters (6), its diagnosis however, continues to be a challenge. Because the clinical and biological features are not yet disease specific, the diagnosis is usually made only by ruling out infection (especially sepsis), autoimmune and neoplastic disease (1, 7). Our previous studies reported that among 61 patients, some patients exhibited a “chronic disease course” or a “poor prognosis,” and 6 patients died of infection, liver failure, and macrophage activation syndrome (8). Therefore, timely diagnosis and accurate monitoring of AOSD activity are essential to improve the quality of therapy. However, the identification of biomarkers with high specificity and sensitivity to diagnosis of AOSD is still lacking.

Recent studies have indicated that circulating microRNAs (miRNAs) have the potential to serve as novel, non-invasive biomarkers for varieties of diseases such as systemic lupus erythematosus (SLE), anti-phospholipid syndrome, organ transplant rejection, and cancers (9–12). miRNAs are sets of small, non-coding RNAs that usually negatively regulate gene expression by inducing messenger RNA (mRNA) cleavage or translational repression (13). miRNAs have been recognized as crucial immune regulators by modulating various phases of inflammation, and their dysregulation has been described in varieties of autoimmune and auto-inflammatory disease, including rheumatoid arthritis, SLE and inflammatory bowel disease (14–16). Emerging evidences have demonstrated that miRNAs in plasma, serum, and other body fluids can be protected from endogenous RNase activity, presenting a stable form in body fluids (17). Therefore, plasma miRNAs may shed a new light on the diagnosis of AOSD.

Previous study has revealed that the expression level of miR-134 in plasma from AOSD patients was increased, and was also correlated with systemic score (18). The diagnostic power of miRNAs in AOSD, however, remains underexplored. In this study, we sought to identify certain plasma miRNAs as non-invasive biomarkers in diagnosis of AOSD using miRNAs deep sequencing technology. Based on the sequencing results and statistical analyses, we prioritized miRNAs. The expression levels of the top 5 upregulated miRNAs (miR-142-5p, miR-101-3p, miR-29a-3p, miR-29c-3p, and miR-141-3p) from miRNAs sequencing results were validated by qRT-PCR. We further investigated the correlations of the miRNA levels with clinical manifestations and inflammatory cytokines.

## Materials and Methods

### AOSD Patients and Healthy Controls Subjects

A total of 100 AOSD patients (74 active and 26 inactive) and 60 healthy controls (HCs) were included in the training and validation sets. An additional independent set consisting of 21 patients with sepsis, 25 AOSD patients and 18 HCs was used to determine the specificity of miRNAs signature in AOSD patients. All AOSD patients fulfilled Yamaguchi's criteria after exclusion of those with infectious, neoplastic and autoimmune disorders (19). All sepsis patients fulfilled the Third International Consensus Definitions for Sepsis and Septic Shock (Sepsis-3) (20). All HCs subjects were recruited from age- and sex-matched volunteers with no history of autoimmune, rheumatic, or other diseases. Information on demographic and clinical data was entered into a database along with the laboratory test results. The AOSD disease activity of each patient was assessed using a modified Pouchot score (21). The experimental design was approved by the Ethics Committee of Ruijin Hospital (identifier 2016-62) and Huashan hospital (identifier 2017-338), Shanghai China, and all the participants provided informed consent. All plasma and serum samples were stored at −80°C immediately after collection.

### miRNA Sequencing and Data Analysis

Data of the miRNA sequencing from the PBMC of AOSD patients and HCs were acquired as previously described (22). PBMC from patients with AOSD (*N* = 3) without any treatment and sex- and age-matched HCs (*N* = 3) were used. In the discovery stage, 482 miRNAs were detected for the purpose of finding the upregulated and downregulated miRNAs.

### Target Gene Prediction and Integrated Analysis by Ingenuity Pathway Analysis

Target genes of the top 5 upregulated miRNAs were predicted and further analyzed to obtain information about biological functions, pathways and networks by using the web-based bioinformatics tool QIAGEN's Ingenuity Pathway Analysis (IPA; Ingenuity Systems, http://www.INGENUITY.com).

### RNA Extraction From Plasma

Total RNAs were extracted from 200 uL of plasma using the QIAzol miRNeasy Serum/Plasma kit (Qiagen, Valencia, CA, USA) according to the manufacturer's instructions. *Caenorhabditis elegans* miRNA (cel-miR-39: 5′-UCACCGGGUGUAA-AUCAGCUUG-3′) was used as a spiked-in control (Qiagen, Valencia, CA, USA). 5.25 × 10^8^ copy of cel-miR-39 was added to each denatured sample after combining the plasma sample with 1 mL QIAzol Lysis Reagent. Total RNAs were eluted with 14 µL of RNase-free water and stored at −80 °C.

### qRT-PCR Analysis of Plasma miRNAs

The cDNA was prepared with the miScript II RT kit (Qiagen) following the manufacturer's instructions. Plasma miRNAs were quantified using miScript SYBR Green PCR Kit (Qiagen) as previously described (10). The relative expression levels of miRNAs were qualified using the equation: amount of target miRNA expression = 2^−Δ*Ct*^, ΔCt = Ct plasma miRNAs- Ct cel-miR-39. The results were magnified 1,000 times.

### Determination of Serum Cytokine

Serum levels of TNF-α, IL-1β, IL-6, and IL-18 were measured by the Meso Scale Discovery electrochemiluminescence assay (MSD, Rockville, MD, USA) according to manufacturer's instructions.

### Statistical Analysis

All data were statistically analyzed using the SPSS version 20.0 software (SPSS Inc., Chicago, IL, USA). Quantitative data were expressed as the means ± SD. Data between two groups with a Gaussian distribution were analyzed using an unpaired *t*-test, while non-parametric data were assessed using the Mann-Whitney *U* test. Data among three groups or more were analyzed using ANOVA (one-way analysis of variance) or Wilcoxon rank-sum test. Receiver operating characteristic (ROC) curves and the area under the ROC curve (AUC) were used to assess the sensitivity and specificity of miRNA biomarkers for the diagnosis of AOSD. We used three different methods (logistic regression, k-nearest neighbor and support vector machine) to find the best combination of the 5 selected miRNAs (miR-142-5p, miR-101-3p, miR-29c-3p, miR-29a-3p, and miR-141-3p) to diagnosis of AOSD. In the training stage, 109 samples (68 AOSD, 41 HCs) were included. In the validation stage, 51 samples (32 AOSD, 19 HCs) were used to verify the model obtained before by means of ROC curve and AUC index. Spearman correlation analysis was performed to test whether the expression levels of 5 miRNAs were correlated with clinical manifestations. *P* < 0.05 was considered statistically significant.

## Results

### Clinical Characteristics of AOSD Patients

Plasma and sera from 100 AOSD patients (74 active and 36 inactive) and 60 healthy controls (HCs) were collected. The clinical characteristics of these subjects in each group are detailed in Table 1. Among the 74 active AOSD patients in the qRT-PCR analysis, common manifestations included spiking fever (73, 100%), sore throat (46, 62.6%), evanescent rash (62, 83.8%), and arthralgia (31, 41.9%). Hepatomegaly, splenomegaly and lymphadenopathy were noted in 5 (6.8%), 36 (48.6.0%), and 58 (78.4%) patients, individually. There were no significant differences in terms of mean age or sex distribution between AOSD patients and HCs.

**Table 1:** Demographic and clinical characteristics of individuals with AOSD.

**Characteristics**	**Active AOSD (*N* = 74)**	**Inactive AOSD (*N* = 26)**	**HCs (*N* = 60)**
Age (years)	36.2 ± 15.6	39.2 ± 11.0	36.1 ± 10.3
Gender (F/M)	60/14	18/8	45/15
Fever	74 (100)	0	/
Sore throat	46 (62.2)	2(7.7)	/
Evanescent rash	62 (83.8)	2(7.7)	/
Arthralgia	31(41.9)	2(7.7)	/
Pneumonia	23 (31.1)	0	/
Pericarditis	17 (23)	0	/
Hepatomegaly	5 (6.8)	0	/
Splenomegaly	36 (48.6)	0	/
Lymphadenopathy	58 (78.4)	0	/
Myalgia	32 (43.2)	0	/
Hemoglobin, g/L	112.0 ± 19.8	115.1 ± 24.4	/
Leukocytes, 10^9^/L	15.2 ± 7.7	10.6 ± 4.2	/
Platelets, 10^9^/L	270.4 ± 112.4	235.2 ± 75.9	/
Ferritin, ng/mL	>2000	329.5 ± 321.1	/
ESR, mm/h	67.1 ± 38.2	19.1 ± 11.7	/
CRP, mg/L	70.2 ± 72.4	9.9 ± 14.2	/
Systemic score	5.8 ± 2.6	0.1 ± 0.3	/

*All values are presented as numbers (with percentage) or mean ± SD (standard deviation)*.

### Differentially Expressed miRNAs in PBMC of AOSD Patients and HCs

**Figure 1:**
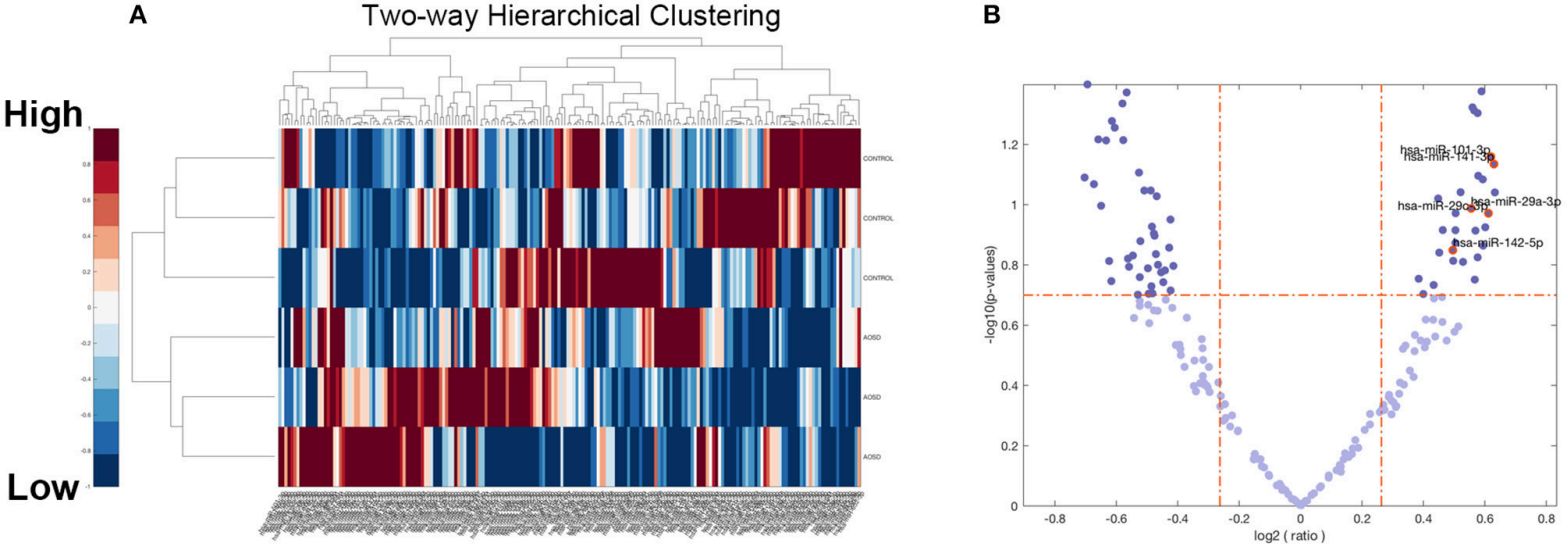
Overview of miRNAs Sequencing Data from AOSD patients. **(A)** Volcano plot of the 482 miRNAs of 6 samples (3 AOSD and 3HCs). The abscissa represents log2 (fold change) of the miRNA expression while the ordinate represents the logarithmic transformation of the *P*-value gained by *t*-test. **(B)** Heat map with two-dimensional clustering of the imputed and normalized miRNA expression in the 6 samples. Missing data are intercepted before this procedure.

To identify miRNAs that were differentially expressed in AOSD and HCs, we per- formed miRNA deep sequencing from PBMC of 3 AOSD patients and 3 HCs. A volcano plot and a heat map (Figure 1) were used to visualize the distinction between the miRNA expressions. Missing data were abandoned or imputed using the nearest-neighbor method, and z-score and 0~1 normalization methods were further conducted on the miRNA expression data. A total of 482 miRNAs were identified in both groups (Figure 1A). We performed filtering between AOSD patients and HCs, using the following criteria: fold change ≥2.0 (or ≤ 0.5) and *P* ≤ 0.05. A hierarchical clustering of differentially expressed miRNAs was shown in Figure 1B, including 10 upregulated miRNAs and 10 downregulated miRNAs, indicating that the expression panels of miRNAs in PBMC of AOSD patients differ from those in HCs. Among the 10 candidate upregulated miRNAs, 5 plasma miRNAs (miR-142-5p, miR-101-3p, miR-141-3p miR-29a-3p, and miR-29c-3p) were significantly increased in AOSD patients (*N* = 20) compared with HCs (*N* = 12) (Supplementary Figure 1). We chose these 5 miRNAs for further analysis.

### Bioinformatic Analysis of Upregulated miRNAs

The five upregulated mature miRNAs were searched in the QIAGEN's Ingenuity Pathway Analysis (IPA) to identify the possible mRNA targets involved in the pathophysiology of AOSD. Three of the interested miRNAs (miR-142-5p, miR-101-3p, miR-29a-3p) were included in the IPA database. The network elaborating the interactions between miRNAs and mRNAs was generated by using the miRNAs Target Filter, filtered by activation of leukocytes, systemic autoimmune disease, viral infection and lymphoma. Confidence was restricted to experimentally observed and highly predicted while species was restricted to human beings (Supplementary Figure 2).

### Circulating miRNA Signature and a Diagnostic Panel in AOSD Patients

**Figure 2:**
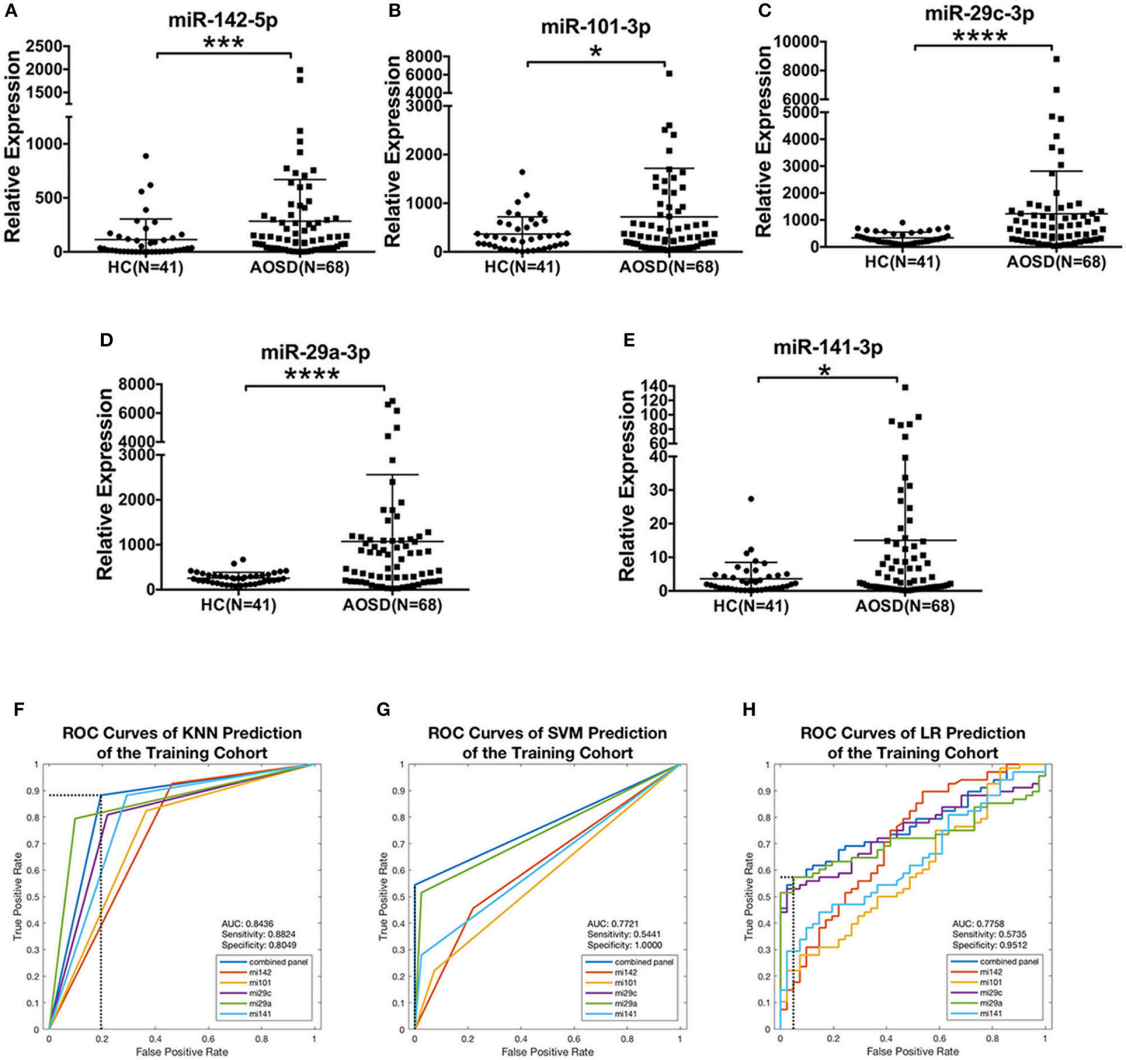
Differentially expressed plasma miRNAs signature for AOSD diagnosis in a training set. Expression levels of **(A)** miR-142-5p, **(B)** miR-101-3p, **(C)** miR-29c-3p, **(D)** miR-29a-3p, **(E)** miR-141-3p were analyzed by qRT-PCR in plasma from AOSD (*N* = 68) and HCs (*N* = 41). The results of histograms represent the means ± SD, ^*^P < 0.05, ^***^P < 0.001, ^****^P < 0.0001. **(F-H)** ROC analysis for individual miRNAs and combined miRNA panel was analyzed by KNN **(F)**, SVM **(G)**, and LR **(H)** analysis.

To confirm the results of miRNA sequencing and hypothesis whether circulating miRNAs secreted by PBMC be a diagnostic tool, we then measured the levels of 5 top upregulated miRNAs (miR-142-5p, miR-101-3p, miR-29c-3p, miR-29a-3p, and miR-141-3p) in plasma in a training set consisting of 68 AOSD patients and 41 HCs. Compared with HCs, we identified a significantly higher level of these 5 miRNAs in plasma from AOSD patients (Figures 2A–E, *P* < 0.001, *P* < 0.05, *P* < 0.0001, *P* < 0.0001, and *P* < 0.05, respectively).

We further accessed the sensitivity and specificity of these 5 miRNAs in diagnosing AOSD using ROC analysis. And then, we used logistic regression to determine the best combination of miRNAs to predict AOSD. Three classification algorithms were used to generate the predictive miRNA combinations: k-nearest neighbor (KNN, *k* = 3), support vector machine (SVM) and binary and multivariate logistic regression model (LR). We found that a linear combination of the expression levels of miR-142-5p, miR-101-3p, and miR-29a-3p generated the best predictive model to diagnose AOSD by using KNN, SVM, and LR. The ROC curves and diagnostic accuracies of three models were shown in Figures 2F–H, and sensitivity and specificity of the classification model were selected by maximizing the Youden index. Compared with the single miRNA, a 3-miRNA panel increased the AUC value to 0.8436, 0.7721, and 0.7758 by KNN, SVM and LR, respectively (Figures 2F–H). The sensitivity was 0.8824 and specificity was 0.8049 by KNN prediction. Taken together, our results suggest that this 3-miRNA panel can be adopted as a diagnostic tool for AOSD.

### Validation of the miRNA Signature for AOSD

**Figure 3:**
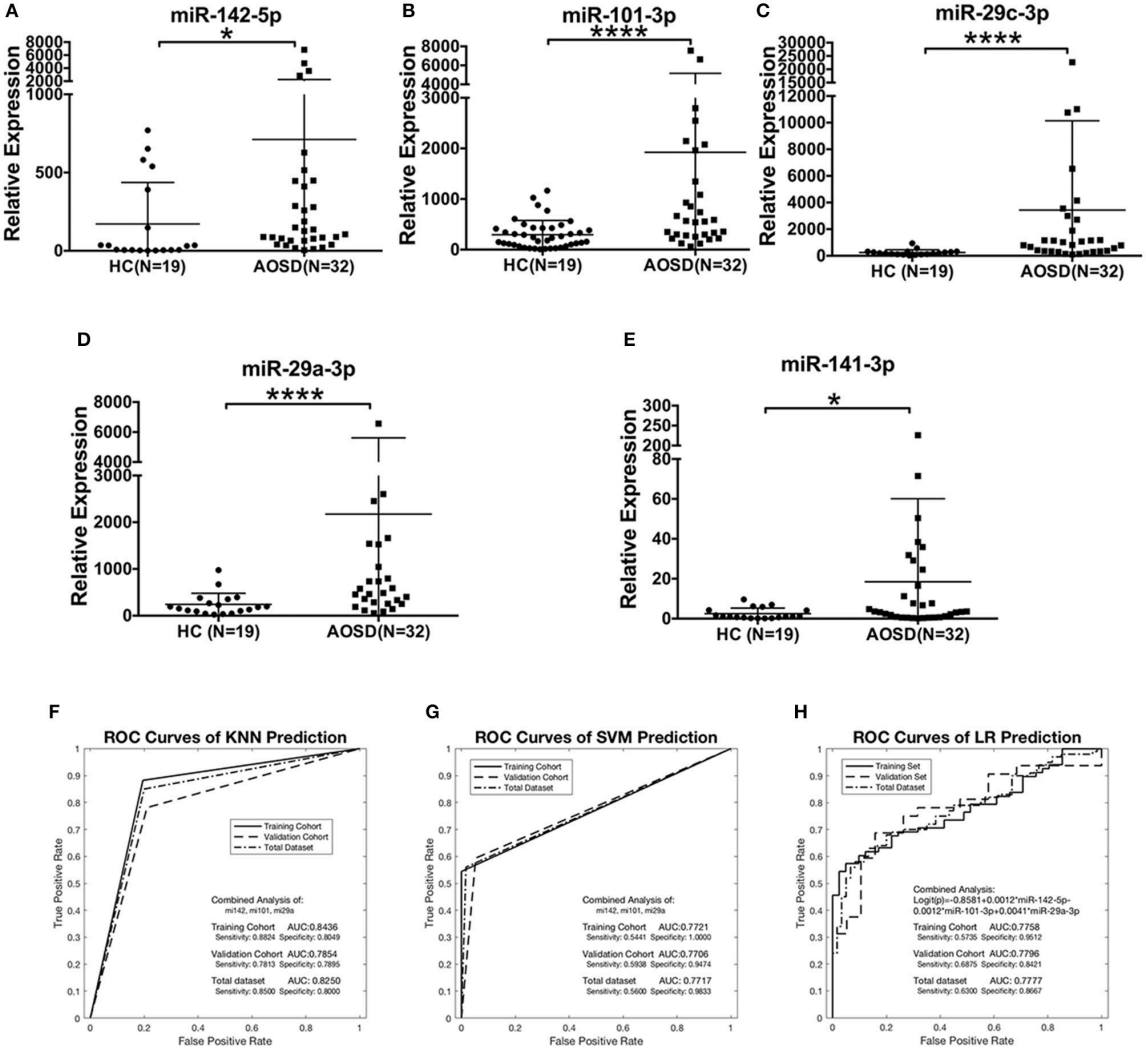
The plasma miRNAs panel for AOSD diagnosis in a validation set. Expression levels of **(A)** miR-142-5p, **(B)** miR-101-3p, **(C)** miR-29c-3p, **(D)** miR-29a-3p, **(E)** miR-141-3p were analyzed by qRT-PCR in plasma from AOSD (*N* = 32) and HCs (*N* = 19). The results of histograms represent the means ± SD, ^*^P < 0.05, ^****^P < 0.0001. **(F-H)** ROC analysis for individual miRNAs and combined miRNA panel was analyzed by KNN **(F)**, SVM **(G)**, and LR **(H)** analysis. Logistic regression demonstrated that a linear combination of values for miR-142-5p, miR-101-3p, and miR-29a-3p produced the best model for AOSD diagnosis.

We further validated the expression levels of 5 miRNAs in an independent validation study consisting of 32 AOSD patients and 19 HCs. The upregulation of 5 miRNAs in plasma of AOSD patients was validated in the validation set (Figures 3A–E). Moreover, we confirmed that the combination of miR-142-5p, miR-101-3p, and miR-29a-3p expression generated the best predictive model in the validation set. The AUC value for the 3-miRNA biomarker panel including miR-142-3p, miR-101-3p, and miR-29a-3p was 0.7854, 0.7706, and 0.7796 by KNN, SVM and LR analysis, respectively (Figures 3F–H). Furthermore, analysis of the data from all 160 samples in training and validation sets produced an AUC value of 0.8250, 0.7717, and 0.7777 by three classification algorithms (Figures 3F–H). And the combination of 3 miRNAs (miR-142-5p, miR-101-3p, miR-29a-3p) generates the best predictive model. Logistic regression produced a model: Logit(p) = −0.8581+0.0012 × miR-142-5p-0.0012 × miR-101-3p +0.0041 × miR-29a-3p (Figure 3H). In a conclusion, our data suggest that the 3-miRNA panel can serve as potential non-invasive biomarkers for AOSD diagnosis.

### The miRNA Signature Was Higher in Active AOSD Patients

**Figure 4:**
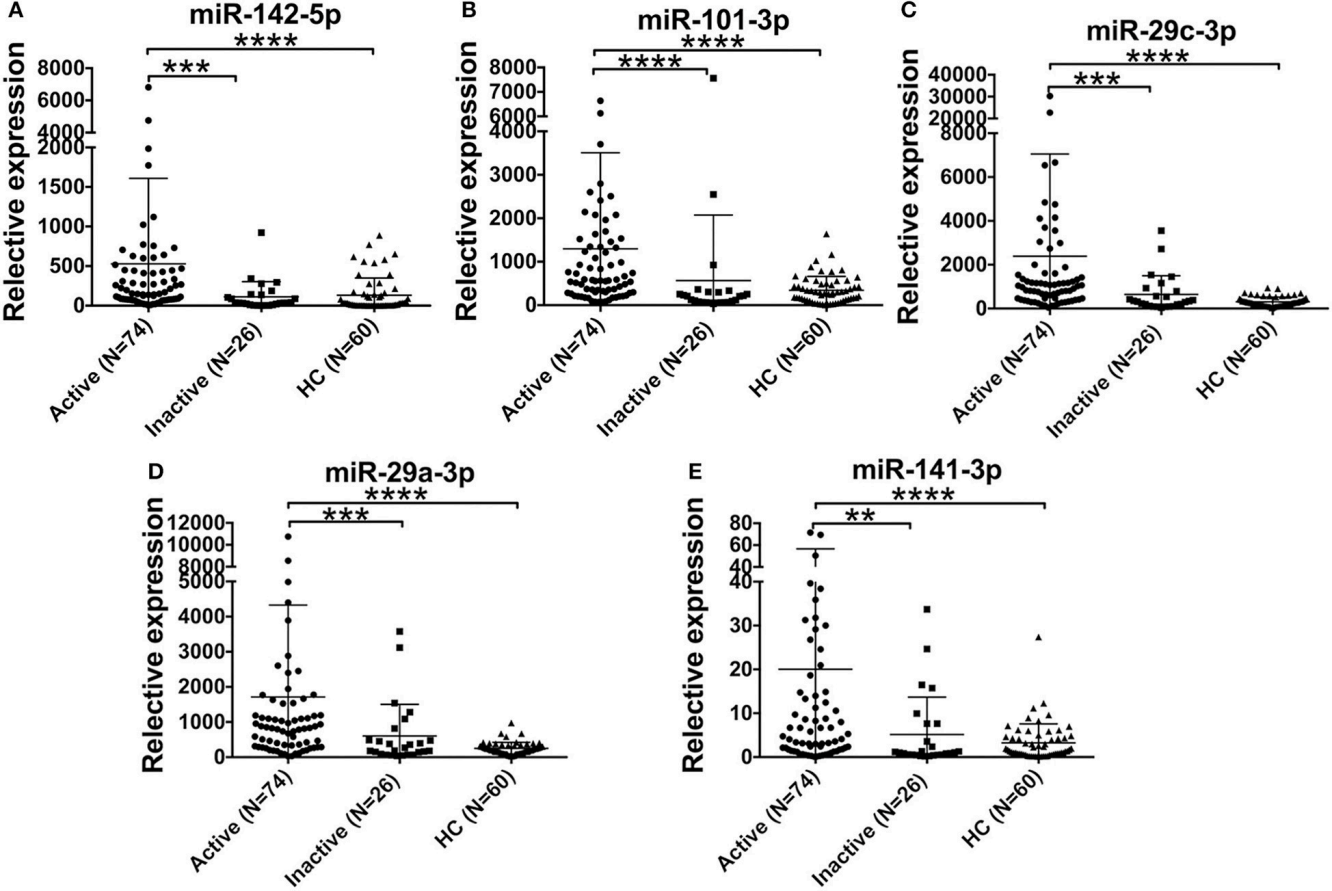
Association of the miRNA signature with AOSD disease activity. Expression levels of **(A)** miR-142-5p, **(B)** miR-101-3p, **(C)** miR-29c-3p, **(D)** miR-29a-3p, **(E)** miR-141-3p in active AOSD (*N* = 74) and inactive AOSD (*N* = 26) patients, and plasma samples from 60 HCs were used as control. The results of histograms represent the means ± SD, ^**^P < 0.01, ^***^P < 0.001, ^****^P < 0.0001.

Furthermore, we compared the levels of 5 miRNAs in AOSD patients with diverse disease activity. The plasma levels of miR-142-5p, miR-101-3p, miR-29a-3p, miR-29c-3p, and miR-141-3p were significantly higher in AOSD patients with active disease (*N* = 74) compared with those with inactive disease (*N* = 26) (Figure 4, *P* < 0.001, *P* < 0.001, *P* < 0.001, *P* < 0.001, and *P* < 0.01, respectively). Moreover, the levels of 5 miRNAs were significantly higher compared with those in HCs. And there was no significant difference in the levels of 5 plasma miRNAs between AOSD patients with inactive disease and HCs. Taken together, our data indicate that plasma miRNA signature is associated with the disease activity of AOSD.

### Circulating miR-101-3p Is Associated With AOSD Clinical Manifestations

We next assessed the correlations between the levels of plasma miRNAs and clinical manifestations. Compared with AOSD patients without high spiking fever, the level of plasma miR-101-3p was significantly higher in AOSD patients with fever (Table 2, *P* = 0.0056). In addition, our results showed that patients with sore throat had higher level of miR-101-3p (Table 2, *P* = 0.0061). And in the presence of arthralgia, the level of miR-101-3p was significantly increased in AOSD patients (Table 2, *P* = 0.0484). Furthermore, the level of miR-29c-3p was significantly higher in patients with myalgia than in patients without myalgia (Table 2, *P* = 0.0143). Thus, miR-101-3p would be considered a potential biomarker to diagnosis AOSD patients timely, as Yamaguchi's criteria includes the clinical manifestations of high spiking fever and arthralgia.

**Table 2:** Comparison of plasma miRNA levels according to disease manifestations in 100 patients with AOSD.

**Manifestations**	**miRNA relative expression levels**		
	**(+), N**	**(–), N**	***P* value**
Fever	*N* = 73	*N* = 27
miR-142-5p	471.83 ± 1082.13	282.41 ± 410.61	0.9896
miR-101-3p	1354.45 ± 2353.83	434.53 ± 579.47	0.0056
miR-29c-3p	1982.41 ± 4617.52	1801.99 ± 2250.34	0.9133
miR-29a-3p	1423.47 ± 2502.69	1427.89 ± 1879.65	0.8733
miR-141-3p	14.63 ± 32.88	20.26 ± 31.18	0.8733
Sore throat	*N* = 48	*N* = 52	
miR-142-5p	415.41 ± 817.68	431.22 ± 1079.02	0.5464
miR-101-3p	1393.12 ± 2223.38	841.10 ± 1901.39	0.0061
miR-29c-3p	1546.18 ± 2345.18	2291.41 ± 5230.19	0.9625
miR-29a-3p	1415.47 ± 2168.01	1433.19 ± 2515.35	0.6073
miR-141-3p	10.75 ± 17.21	21.14 ± 41.33	0.3228
Skin rash	*N* = 64	*N* = 36	
miR-142-5p	491.97 ± 1151.57	293.96 ± 376.25	0.6337
miR-101-3p	1396.86 ± 2495.26	589.13 ± 697.69	0.089
miR-29c-3p	2218.77 ± 4908.61	1426.90 ± 1964.39	0.6068
miR-29a-3p	1573.35 ± 2650.66	1160.33 ± 1658.41	0.379
miR-141-3p	15.81 ± 35.67	16.77 ± 25.95	0.6384
Arthralgia	*N* = 31	*N* = 69	
miR-142-5p	482.05 ± 1109.87	284.11 ± 400.08	0.7076
miR-101-3p	1387.21 ± 1136.99	480.31 ± 549.51	0.0484
miR-29c-3p	2054.47 ± 4746.24	1664.88 ± 2100.68	0.6828
miR-29a-3p	1464.63 ± 2572.78	1335.70 ± 1754.67	0.8638
miR-141-3p	15.06 ± 33.79	18.59 ± 29.35	0.437
Hepatomegaly	*N* = 5	*N* = 95	
miR-142-5p	233.75 ± 201.34	430.53 ± 973.35	0.9755
miR-101-3p	787.84 ± 664.17	1122.82 ± 2118.88	0.9145
miR-29c-3p	1077.73 ± 687.18	1978.75 ± 4204.49	0.6784
miR-29a-3p	949.62 ± 698.57	1449.67 ± 2396.11	0.7241
miR-141-3p	6.46 ± 5.27	16.67 ± 33.13	0.9897
Splenomegaly	*N* = 36	*N* = 64	
miR-142-5p	519.53 ± 1236.88	365.09 ± 748.63	0.2874
miR-101-3p	1539.87 ± 2586.83	867.13 ± 1690.15	0.0685
miR-29c-3p	2660.81 ± 6197.49	1524.70 ± 2158.06	0.5403
miR-29a-3p	1791.97 ± 3133.98	1218.05 ± 1743.35	0.5031
miR-141-3p	14.84 ± 37.90	16.89 ± 29.10	0.4057
Lymphadenopathy	*N* = 58	*N* = 42	
miR-142-5p	449.77 ± 1014.04	380.52 ± 864.96	0.1953
miR-101-3p	1244.51 ± 2132.3	914.90 ± 1990.92	0.1359
miR-29c-3p	2097.23 ± 5000.84	1707.87 ± 2410.76	0.9183
miR-29a-3p	1466.15 ± 2618.02	1367.37 ± 1924.86	0.9791
miR-141-3p	15.73 ± 35.54	16.74 ± 27.82	0.544
Pericarditis	*N* = 17	*N* = 83	
miR-142-5p	442.79 ± 846.72	416.16 ± 974.82	0.4496
miR-101-3p	1343.04 ± 2268.19	1057.54 ± 2038.83	0.8058
miR-29c-3p	2600.20 ± 5751.09	1797.19 ± 3710.82	0.4846
miR-29a-3p	1603.00 ± 2884.92	1388.14 ± 2233.37	0.3585
miR-141-3p	17.98 ± 34.11	15.78 ± 32.21	0.5556
Pleuritis	*N* = 23	*N* = 77	
miR-142-5p	496.94 ± 897.48	397.91 ± 970.00	0.3198
miR-101-3p	1664.74 ± 3108.67	939.20 ± 1631.92	0.5412
miR-29c-3p	2550.14 ± 5302.76	1749.57 ± 3694.73	0.6445
miR-29a-3p	1671.38 ± 2919.52	1350.97 ± 2156.66	0.4957
miR-141-3p	18.75 ± 31.85	15.38 ± 32.69	0.2311
Pneumonia	*N* = 23	*N* = 77	
miR-142-5p	496.94 ± 897.48	397.91 ± 970.00	0.3198
miR-101-3p	1664.74 ± 3108.67	939.20 ± 1631.92	0.5412
miR-29c-3p	2550.14 ± 5302.76	1749.57 ± 3694.73	0.6445
miR-29a-3p	1671.38 ± 2919.52	1350.97 ± 2156.66	0.4957
miR-141-3p	18.75 ± 31.85	15.38 ± 32.69	0.2311
Myalgia	*N* = 32	*N* = 68	
miR-142-5p	191.25 ± 245.06	528.66 ± 1126.77	0.1195
miR-101-3p	791.38 ± 1136.99	1254.17 ± 2380.24	0.8942
miR-29c-3p	689.19 ± 599.97	2519.35 ± 4861.38	0.0143
miR-29a-3p	674.59 ± 792.04	1777.64 ± 2723.32	0.0517
miR-141-3p	7.68 ± 14.15	20.14 ± 37.49	0.0969

### Circulating miR-101-3p Is Associated With Serum Levels of IL-6 and TNF-α

**Figure 5:**
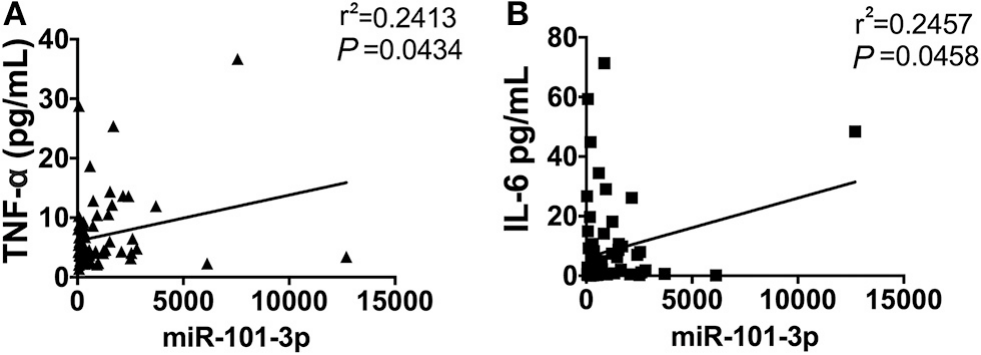
The correlation of miR-101-3p with serum IL-6 and TNF-α. Correlation analysis of miR-101-3p with serum levels of **(A)** IL-6 and **(B)** TNF-α in AOSD patients.

It has been demonstrated that inflammatory cytokines play a critical role in the pathogenesis of AOSD, we next measured the correlation of plasma miRNAs levels with inflammatory cytokines, including IL-1β, IL-18, IL-6, and TNF-α. As shown in Figure 5, the level of plasma miR-101-3p was significantly positively correlated with TNF-α (*r*^2^ = 0.2413, *P* = 0.0434) and IL-6 (*r*^2^ = 0.2457, *P* = 0.0458) in serum. We found that other miRNA levels showed no significant correlation with IL-1β, IL6, IL-18, and TNF-α in AOSD patients (data were not shown). In a conclusion, the results indicate that miR-101-3p may play an important role in the expression levels of IL-6 and TNF-α.

### Potential Value of miRNA Signature in the Differential Diagnosis of AOSD From Sepsis

**Figure 6:**
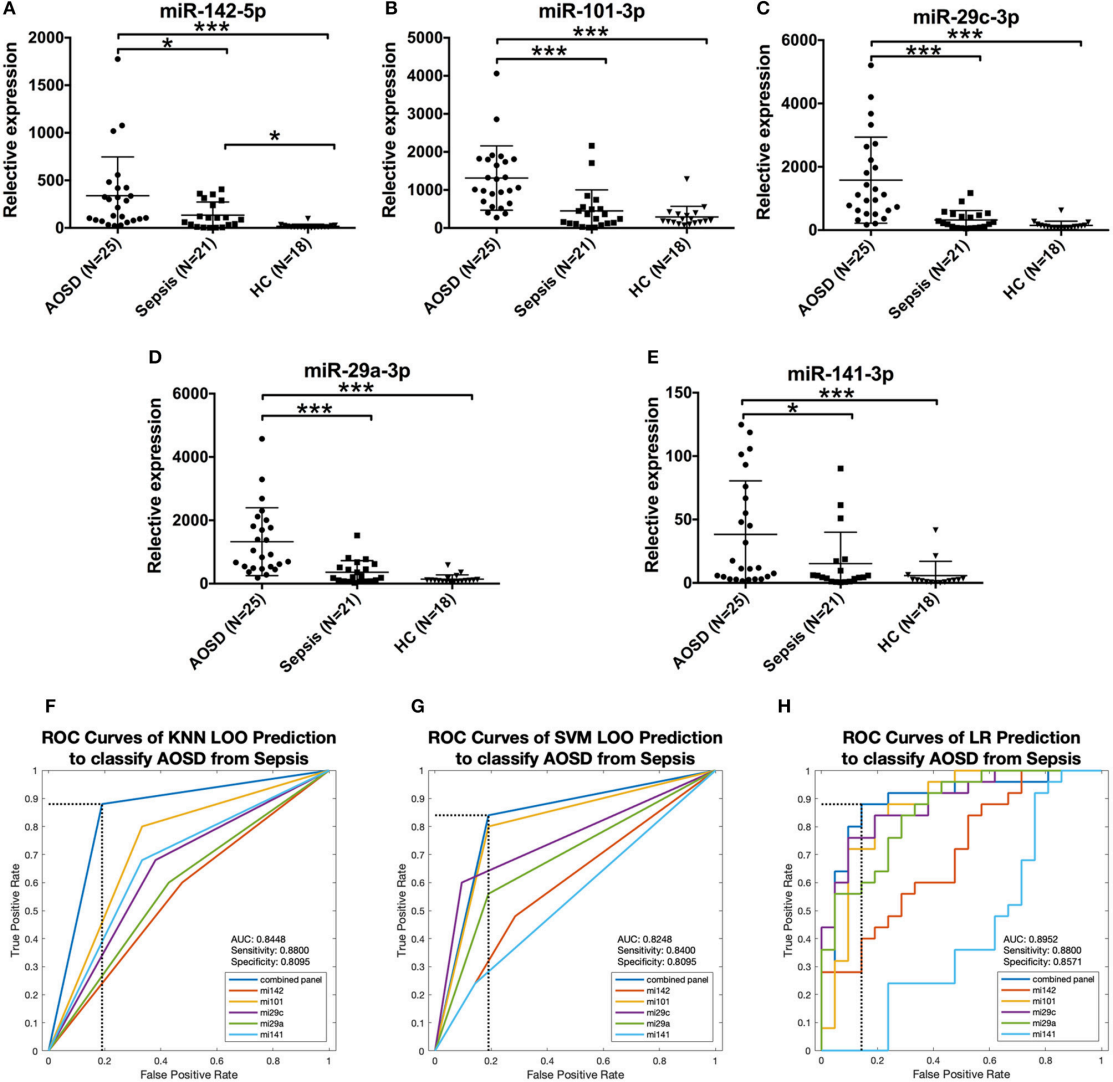
Expression of AOSD miRNA signature in sepsis patients. Expression levels of **(A)** miR-142-5p, **(B)** miR-101-3p, **(C)** miR-29c-3p, **(D)** miR-29a-3p, **(E)** miR-141-3p were analyzed by analyzed by qRT-PCR in an independent set consisting of HC (*N* = 18), AOSD (*N* = 25) and sepsis (*N* = 21) patients. The results of histograms represent the means ± SD, ^*^P < 0.01, ^***^P < 0.001. **(F-H)** ROC analysis for individual miRNAs and combined miRNA panel was analyzed by KNN **(F)**, SVM **(G)** and LR **(H)** analysis. Logistic regression demonstrated that a linear combination of values for miR-142-5p, miR-101-3p, miR-29c-3p, and miR-141-3p produced the best model to distinguish AOSD patients from sepsis patients.

It's very important and difficult to differentiate sepsis from AOSD in clinical practice. To determine the disease specificity of the 5 miRNAs identified in AOSD patients, we enrolled patients with sepsis (*N* = 21) as non-AOSD disease controls. Samples from an independent set consisting of AOSD patients (*N* = 25) and HCs (*N* = 18) were collected. The expression levels of 5 miRNAs (miR-142-5p, miR-101-3p, miR-29c-3p, miR-29a-3p, and miR-141-3p) were significantly higher in AOSD patients compared to that in sepsis patients (Figures 6A–E). We further accessed the sensitivity and specificity of 5 miRNAs in distinguishing AOSD from sepsis using ROC analysis. We found that 4-miRNA panel (miR-142-5p, miR-101-3p and miR-29c-3p and miR-141-3p) produced the best predictive model to separate AOSD from sepsis by using KNN, SVM, and LR. Compared with the single miRNA, a 4-miRNA panel increased the AUC value to 0.8448, 0.8248, and 0.8952 by KNN, SVM, and LR, respectively (Figures 6F–H). The diagnostic sensitivity of the panel for AOSD distinguishing from sepsis patients was 0.88 and the specificity was 0.8095 by KNN prediction, and SVM and LR analysis also showed high sensitivity and specificity (Figures 6F–H). Taken together, these data suggest that these 5 miRNAs have a potential value to distinguish AOSD patients from sepsis.

## Discussion

In this study, we identified a set of plasma miRNAs as potential biomarkers to diagnose AOSD and monitor disease activity. We found that 5 miRNAs (miR-142-5p, miR-101-3p, miR-29a-3p, miR-29c-3p, and miR-141-3p) were significantly increased in plasma of AOSD patients when compared with HCs. We developed a model including 3 miRNAs (miR-142-5p, miR-101-3p, and miR-29a-3p) to predict the probability of AOSD. Moreover, the expression levels of these 5 miRNA biomarkers were significantly upregulated in active AOSD patients. Elevated level of miR-101-3p was found in AOSD patients with fever, sore throat and arthralgia symptoms, and miR-101-3p was positively correlated with serum levels of IL-6 and TNF-α. In addition, elevated miRNAs were not found in patients with sepsis, and 4-miRNA panel (miR-142-5p, miR-101-3p, miR-29c-3p, and miR-141-3p) can distinguish AOSD from sepsis with an AUC value of 0.8448. Our results suggest that circulating miRNAs have potential value to separate AOSD patients with sepsis, and evaluate disease activity.

In the new decade, many studies have demonstrated that circulating miRNAs have great potential as novel specific biomarkers for diagnosis disease, including tumor, infectious disease, cardiovascular disease and autoimmune disease (23). The diagnosis of autoimmune diseases is often supported by the presence of autoantibodies (such as antinuclear autoantibodies, RF, ACPA) or autoantigen- specific T cells and B cells (24). However, to diagnosis of AOSD immediately is still a challenge in clinical practice. To date, there is no widely recognized protein biomarker for diagnosis of AOSD. The delay to final diagnosis exists in AOSD. A retrospective observational research found that a mean diagnosis delay was 4 months (25). Evidences have been suggested that high levels of serum IL-18, S100A8/9, S100A12 are found in AOSD patients (3, 24). While these biomarkers are not specific to AOSD and also be elevated in patients with inflammatory and autoimmune disease. High serum level of IL-18 is also observed in sepsis patients (21, 26). And serum levels of S100A8/9 and S100A12 are also elevated in SLE and infectious disease (3, 27–29). Thus, there is indeed a need for identifying highly sensitive and specific biomarkers, which can be used not only to diagnosis of AOSD and monitor disease activity, but also to differentiate AOSD from other inflammatory disease. In our study, we explored the expression levels of miRNAs in AOSD, and investigated diagnostic potential of miRNA signature in AOSD.

To diagnosis of AOSD has to rule out other inflammatory disease, especially sepsis. There are many similarities in clinical manifestations and laboratory tests between AOSD and sepsis (21). It is suggested that hyperferritinemic syndrome includes AOSD and sepsis (30). Moreover, sepsis is a life-threatening disorder and a major concern for human health globally as its high mortality. Conventional diagnostic practices are time consuming and lack of high specificity and sensitivity (31). It is recognized that diagnosis of sepsis timely and rapid administration of antibiotics are associated with lower mortality (32). While the treatments of AOSD include corticosteroid, methotrexate (MTX) and biologic drugs, which are different from sepsis (33). Therefore, the differential diagnosis between AOSD and sepsis is a great challenge and of great importance in clinical practice. Our measurement of the expression levels of the miRNA signature for AOSD clearly demonstrate that it is elevated in the plasma of AOSD patients but is not detected in sepsis. And the AUC value of 4-miRNA panel (miR-142-5p, miR-101-3p, miR-29c-3p, and miR-141-3p) was 0.8448, shedding new light to differentiate AOSD from sepsis.

To the best of our knowledge, circulating miR-142-5p, miR-101-3p, miR-29a-3p, miR-29c-3p, and miR-141-3p have not previously been linked to AOSD. Our study indicates that, the combination of three miRNAs (miR-142-5p, miR-101-3p, and miR-29a-3p) has highest AUC value that distinguishes AOSD group from healthy group with high sensitivity and specificity. Among the studies of miRNAs, miR-142-5p was shown to be upregulated in patients with multiple sclerosis and to promote T cell differentiation toward Th1 cells, leading to accelerate inflammation (34). In another inflammation model, experimental colitis, blocking miR-142-5p expression ameliorates disease by IL-10RA pathway (35). About miR-101-3p, it has been reported to a diagnostic biomarker for identifying heart transplant patients with acute cellular rejection (36). Furthermore, it has been demonstrated that the target of miR-29a-3p is Gab1 (37), which plays an vital role in inflammation by inhibiting activation of NK-κB. The remarkable capability of NK-κB is to trigger inflammation by inducing genes expression, including IL-1β, IL-6 and TNF-α (38, 39). By inhibiting the expression of Gab1, insulin-like growth factor 1 accelerates endothelial cells inflammation and atherosclerosis (40), suggesting that upregulation of miR-29a-3p in AOSD patients could facilitate inflammatory response by decreasing Gab1 expression. These results indicate that the combination of miR-142-5p, miR-101-3p, and miR-29a-3p can be a validated tool to assess the inflammation activity. Consistent with our hypothesis, the level of miR-101-3p was positively correlated with IL-6 and TNF-α in serum. To data, the role of miR-101-3p in regulating IL-6 and TNF-α has to be determined. Future studies should focus on identifying the pathogenic role of these miRNAs in AOSD.

NLRP3 inflammasome, the key inducer of inflammation in response to pathogens and innate immune stimuli, is activated in AOSD patients (41). While the mechanism of how NLRP3 is activated in AOSD remains unclear. Recent publications demonstrate that miR-223 plays a crucial role in inflammation by inhibiting NLRP3 activation (42). Liao et al. found that the level of miR-223 was upregulated in AOSD patients by miRNAs microarray assay (18). However, the upregulation of miR-223 has not been validated by qRT-PCR. They found that the level of miR-134 was significantly higher in plasma from 48 AOSD patients, and positively correlated with disease activity scores and plasma IL-18 levels, indicating that the upregulation of miR-134 may be a potential prognostic biomarker. Nevertheless, little is known about the diagnostic value of circulating miRNAs panel in AOSD. The presence of miR-134 was also found in our miRNA sequencing. Our results indicated that there was no significant difference between AOSD patients and healthy controls. Genetics plays an important role in the pathogenesis of AOSD, there may exist some differentially expressed miRNAs in different populations.

In a conclusion, the miRNAs in plasma were found to be potential non-invasive biomarkers for diagnosis of AOSD. The expression level of miR-101-3p was positively correlated with serum levels of IL-6 and TNF-α, suggesting that miR-101-3p may play a crucial role in inflammatory response in AOSD.

## Ethics Statement

The study was performed in accordance with the Declaration of Helsinki and the principles of Good Clinical Practice. Biological samples were obtained under a protocol approved by the Institutional Research Ethics Committee of Ruijin Hospital (ID: 2016-62) and Huashan Hospital (identifier 2017-338), Shanghai, China. All subjects signed written informed consent.

## Author Contributions

QH participated in experiments, provided the figures, and wrote the manuscript. WG participated in RNA extraction and qRT-PCR. JG performed bioinformatic analysis. GG and TLi helped to revise the manuscript. RT, ZY, HZ, and LS provided the plasma from sepsis patients. TLiu prepared the Table 1 and figures. LW and JJ collected the clinical samples and data. CY designed the experiments and revised the manuscript. YS and HS designed experiments, performed statistical analysis and revised the manuscript. All authors read and approved the final manuscript.

### Conflict of Interest Statement

The authors declare that the research was conducted in the absence of any commercial or financial relationships that could be construed as a potential conflict of interest.

## Abbreviations

AOSD: Adult-onset Still's disease
miRNA: MicroRNA
HCs: healthy controls
ROC, receiver operating characteristic AUC: the area under the ROC curve
ESR: erythrocyte sedimentation
CRP: C-reactive protein
mRNA: massager RNA
SLE: systemic lupus erythematosus
IPA: QIAGEN's Ingenuity Pathway Analysis
cel-miR-39: *Caenorhabditis elegans* miRNA
MTX: methotrexate.
