# Supplementary material for: Plasma microRNA Profiles as a Potential Biomarker in Differentiating Adult-Onset Still's Disease From Sepsis
Source: Front Immunol. 2019 Jan 11;9:3099. doi: 10.3389/fimmu.2018.03099 (PMC6338094; doi:10.3389/fimmu.2018.03099)
Supplement: Supplementary file 1 [file Data_Sheet_1.pdf]

Plasma microRNA profiles as a potential biomarker in differentiating adult-onset still's disease from sepsis

Qiongyi Hu [1†], Wen Gong [1, 2†], Jieyu Gu [1†], Guannan Geng [3], Ting Li [3], Rui Tian [4], Zhitao Yang [5], Haocheng Zhang [6], Lingyun Shao [6], Tingting Liu [1], Liyan Wan [1], Jinchao Jia [1], Chengde Yang [1*], Yi Shi [7*], Hui Shi [1*]

1. Department of Rheumatology and Immunology, Ruijin Hospital, Shanghai Jiao Tong University School of Medicine, Shanghai, China.
2. Department of Rheumatology and Immunology, The First People's Hospital of Yancheng affiliated with Nantong University, Yancheng, China.
3. Department of Rheumatology, Renji Hospital, Shanghai Jiao Tong University School of Medicine, Shanghai, China.
4. Department of Critical Care Medicine, Ruijin Hospital, Shanghai Jiao Tong University School of Medicine, Shanghai, China.
5. Department of Emergency, Ruijin Hospital, Shanghai Jiao Tong University School of Medicine, Shanghai, China.
6. Department of Infectious Diseases, Huashan Hospital, Fudan University, Shanghai, China.
7. Key Laboratory of Systems Biomedicine (Ministry of Education) and Collaborative Innovation Center of Systems Biomedicine, Shanghai Center for Systems Biomedicine, Shanghai Jiao Tong University, Shanghai, China.

***Corresponding authors:**

Chengde Yang

Email: yangchengde@hotmail.com;

Yi Shi

Email: yishi@sjtu.edu.cn

Hui Shi

Email: shihui_sjtu@sina.com;

[†]These authors contributed equally to this work.

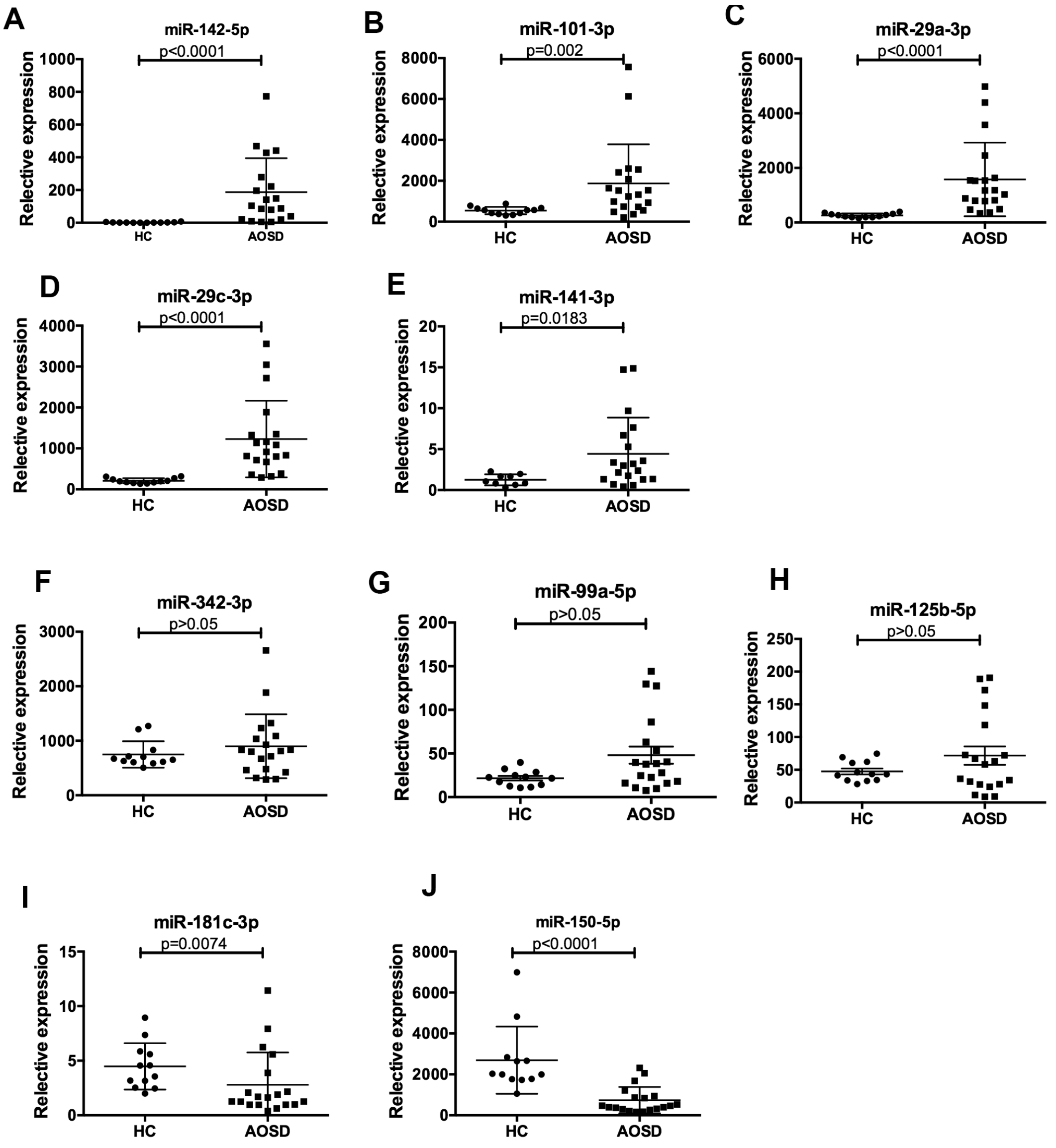

Supplementary Figure 1. The top 10 upregulated miRNAs in plasma of AOSD patients. Expression levels of **(A)** miR-142-5p, **(B)** miR-101-3p, **(C)** miR-29a-3p, **(D)** miR-29c-3p, **(E)** miR-141-3p, **(F)** miR-342-3p, **(G)** miR-99a-5p, **(H)** miR-125b-5p, **(I)** miR-181c-3p and **(J)** miR-150-5p were analyzed by qRT-PCR in plasma from AOSD (N=20) and HCs (N=12). The results of histograms represent the means ± SD.

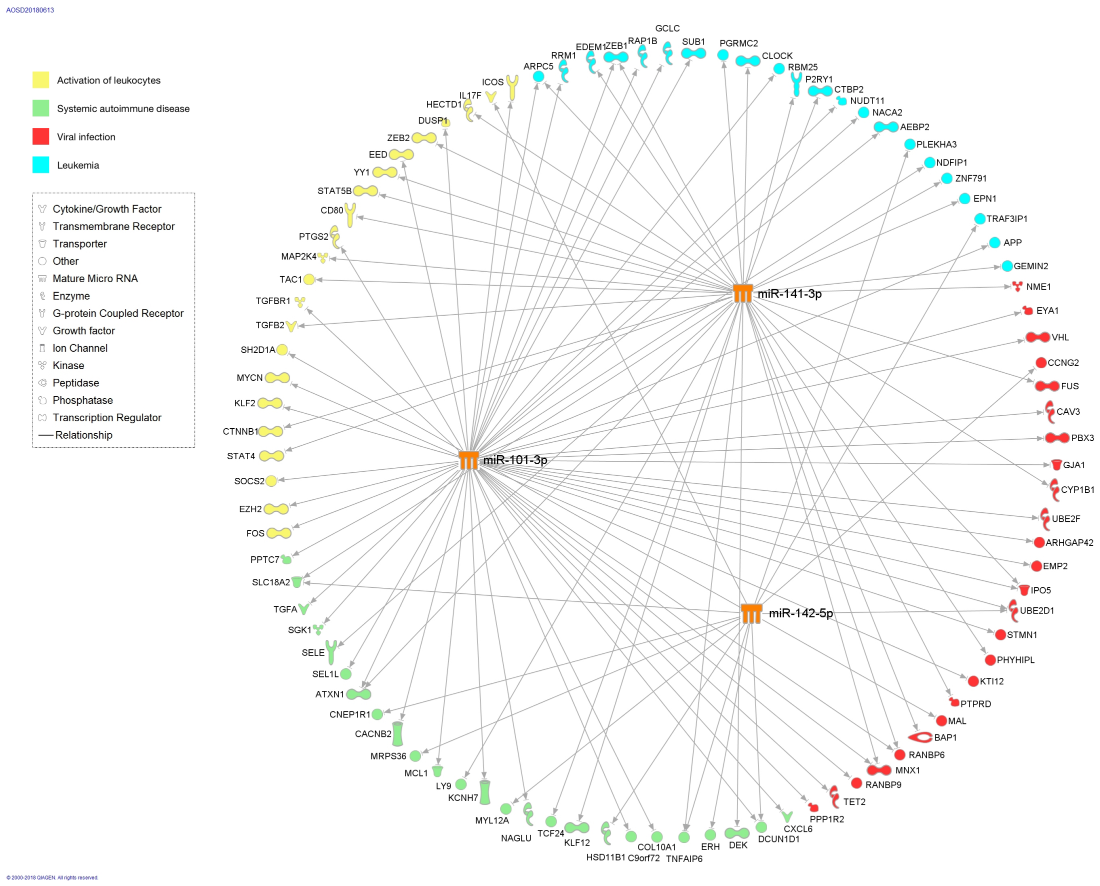

Supplementary Figure 2. Interaction Network of miRNAs identified potential mRNA targets involved in clinical features of AOSD.
